# Ga-Based Liquid Metals: Advances in Interface Thermal and Electrical Regulations for Power Electronics Integration

**DOI:** 10.3390/ma19081599

**Published:** 2026-04-16

**Authors:** Canyu Liu, Tianqi Liu, Zhiwei Huang, Xiangyi Li, Jiabao Zheng, Guoxi Li, Gan Wang, Wentao Liu, Changqing Liu

**Affiliations:** 1 School of Mechanical Science and Engineering, Huazhong University of Science and Technology, Luoyu Road 1037, Wuhan 430074, China; 2 School of Materials Science and Engineering, Huazhong University of Science and Technology, Luoyu Road 1037, Wuhan 430074, China

**Keywords:** Ga-based liquid metals, power electronics integration, interfacial reaction, metallization, ultrasonic vibration

## Abstract

Ga-based liquid metals (GLMs) have been considered as promising thermal and electrical interface materials for advanced power electronics, combining high thermal conductivity (some types even >30 W/m·K) with fluidity at room temperature. This review systematically evaluates the dual roles of GLMs in power electronics packaging. Their function in thermal management as both thermal interface materials and active cooling media is first examined, followed by an analysis of their capabilities in forming electrical interconnections via low-temperature bonding in fluidic and solid states. However, reliable integration remains challenging due to interfacial reactions and instability with metal substrates. We discuss interfacial mechanisms with Cu and common metallizations, along with emerging regulation strategies such as surface coatings and process acceleration techniques. By examining these interfacial interactions, this work aims to guide the selection and design of surface modification strategies to either promote or inhibit reactions as needed, supporting the development of robust power electronic packaging.

## 1. Introduction

### 1.1. Recent Progress in Power Electronics Packaging

Driven by quick developments in the electric vehicle, aerospace and energy industry, there is a growing need for semiconductors with superior operational performance and reliability [1]. To meet these demands, wide bandgap (WBG) semiconductors, including SiC and GaN, have emerged as promising candidates. They are widely regarded as the next-generation electronics, poised to replace conventional Si devices in high-power applications [2]. The main advantages of WBG devices are enabled by their wide bandgap, which affords them a higher breakdown voltage, faster switching frequency and greatly diminished switching losses [3]. The theoretical maximum operating temperature of WBG semiconductors exceeds 600 °C, which is significantly higher than the upper limit (~200 °C) of conventional Si devices [4]. 

To keep pace with the rapid development of WBG devices, the related packaging systems must meet more stringent mechanical, electrical and thermal requirements [5]. Currently, packaging and integration capabilities are the major bottleneck that limits the full potential of WBG devices [6]. The conventional interconnection materials are not sufficiently stable at elevated temperatures exceeding 200 °C [7]. This is critical in die-attach, which refers to the electrical interconnection between the die and the substrate [8]. Moreover, thermal interface materials (TIMs) play a critical role in power electronic packaging by bridging the thermal paths between the heat-generating chip and heat sink [9]. As heat-flux densities continuously rise in power modules, the properties of TIMs directly dictate their maximum operating performance [10].

Recently, Ga-based liquid metals (GLMs) have emerged as promising materials for both thermal and electrical interfaces in power electronics packaging. GLMs are ideal candidates for TIMs, as they uniquely combine the metal-like thermal conductivity and liquid-like conformability [11]. This enables them to effectively eliminate air gaps and wet rough surfaces spontaneously, thereby minimizing interfacial thermal resistance [12]. At the same time, GLMs enable low-temperature transient liquid-phase bonding (TLPB) by serving as a reactive metallic solvent [13]. Their low melting point allows immediate liquid-phase bonding below regular Sn-based soldering and nano Ag sintering technology [14]. The rapid inter-diffusion with substrate metals forms refractory intermetallic compounds (IMCs) that create high-temperature capable joints [15]. These advantages of low-temperature bonding and high-temperature serving performance make GLMs promising for high-power electronics integration.

### 1.2. Power Electronics Packaging Failure Induced by Packaging Interfaces

The reliability of power electronics highly depends on the packaging interfaces, which are formed by the interfacial reactions during both bonding and serving processes [16]. It has been calculated that 55% of die-attach failures were caused by the cracks that usually initiate at the die/solder interface [17]. Thus, the interfacial reactions during joint formation ultimately determine the failure mechanism and in-service lifetime. The metallization deposited on the surfaces of components is another critical factor as it can highly influence the interfacial reactions and the formation of microstructure [18], thereby altering the failure mechanisms of the joints [19]. 

The coefficient of thermal expansion (CTE) mismatch is a critical challenge to the reliability of power electronics packaging, which usually leads to interfacial failures. Different materials expand or contract at different rates during temperature changes, which develops high thermal stress at the interfaces [20]. These stresses can cause failures such as cracks, delamination and bondline fatigue. In 2.5D packaging, CTE mismatch between the substrate and the printed circuit board is the main reason for the fatigue behavior of board-level ball grid array (BGA) solder joints [21]. In high-power module packaging that uses nano Ag sintering technology, the CTE mismatch between the Ag layer and the die or ceramic substrate causes cracks at the interface edges during thermal cycling [22]. This damages the heat dissipation path and electrical connection [23]. Notably, when fluidic GLMs are applied, the interfacial thermal stress induced by CTE mismatch is expected to be decoupled. Consequently, the associated interfacial failure mechanism may differ, which warrants further investigation.

### 1.3. Scope of Review

Despite the significant research on GLMs for electrical or thermal connections that have been published in recent years, few studies have analyzed the applications and regulation strategies of interfacial reaction to address power electronics reliability. There is a pressing need to bridge the existing knowledge gap to elaborate the thermal and electrical interface, in order to optimize the suitable serving interface by matching specific GLMs. The initial motivation to develop various thermal and electrical interfaces is primarily to address the use of conventional TIMs and interconnection materials. However, the uses of thermal and electrical interfaces are, nowadays, much wider given the new emerging GLMs, which are anticipated to be applicable for the harsh serving environment of high-power electronics.

**Figure 1:**
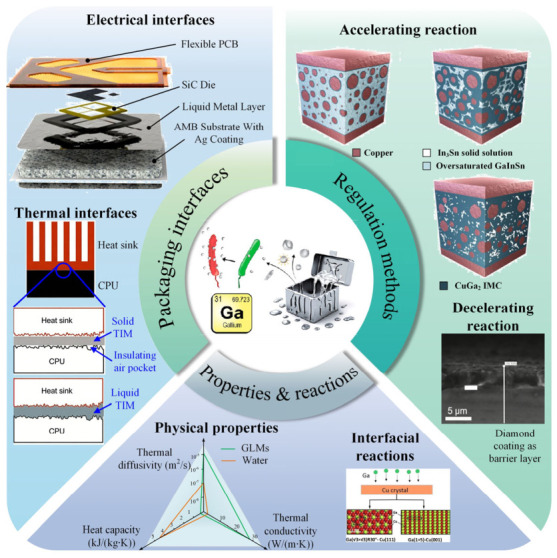
Summary of GLMs applied in power electronics integration [9,11,24,25,26].

In this review paper, the thermal and electrical interfaces of GLMs as well as their regulation methods were reviewed in line with power electronics applications, as shown in Figure 1. Subsequently, the advantages of employing GLMs as thermal and electrical interface materials are analyzed. Motivated by a detailed analysis of interfacial reaction-induced reliability failures in WBG power modules under harsh conditions, effective regulation strategies have been proposed to ensure robust service performance. The accelerating interfacial reaction methods with GLMs were used for the TLPB process to form the electrical interconnection structure. On the other hand, the decelerating interfacial reaction methods with GLMs were applied to maintain stability. Finally, the guidance and recommendations are provided for the optimization of thermal and electrical interfaces applied in future reliable WBG power module integrations.

## 2. Applications of GLMs in Electronics Packaging Interfaces

### 2.1. GLMs Applied on Thermal Interfaces

#### 2.1.1. GLMs Applied as Thermal Interface Materials (TIMs)

**Figure 2:**
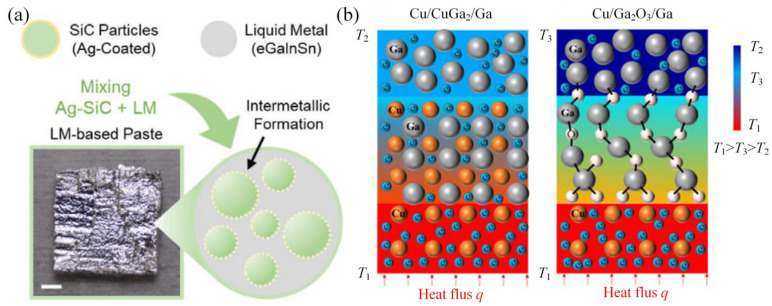
(**a**) Example of GLM applied as TIM; (**b**) schematic of the microscopic interfacial heat transfer mechanism [30,35].

GLMs are regarded as the promising next-generation TIMs for power electronics thermal management [27]. By enhancing interfacial wetting and reducing contact thermal resistance, it can significantly improve the heat dissipation for high-power modules [28]. The excellent fluidity of GLMs enables them to eliminate air gaps, thereby inducing low thermal interface resistance. In addition, GLMs have advantages including self-healing capabilities, high thermal stability and non-flammability, which allow them to serve in harsh thermal interfaces. However, the high mobility of GLMs also faces challenges such as leakage and corrosion when in direct contact with metal heat sinks. Therefore, advanced strategies need to be developed to maintain high thermal conductivity and prevent leakage in the applications of power electronics (Figure 2a).

Liu et al. [29] demonstrated that GLM-based TIMs reduce chip temperature by up to 9.8 °C at 90 W load, outperforming conventional thermal grease. Their study also indicated that thinner TIM layers and larger interface areas enhance heat dissipation. Zhang et al. [30] achieved a significant reduction in thermal boundary resistance at the Ga/Cu interface by forming a CuGa_2_ IMC layer. As illustrated in Figure 2b, this reduction was attributed to the transition of heat carriers from phonons to electrons and the strengthening of interfacial bonding from van der Waals forces to metallic bonds. Wu et al. [27] achieved excellent wetting of GLMs on Cu substrates, forming a stable thermal interface with minimal leakage and attaining a low total thermal resistance of 2.5–3 K·mm^2^/W, which is substantially lower than the conventional thermal greases. Lin et al. [31] conducted a microstructural analysis of GLM interfaces used as TIMs. They showed that heat treatment transformed brittle CuGa_2_ into more stable Cu_9_Ga_4_, thereby improving both joint strength and thermal performance. Chen et al. [12] developed a sandwich-structured thermal pad in which a GLM/Ni-coated Cu core is encapsulated within an insulating polymer shell. This design achieved a high thermal conductivity of 12.41 W/(m·K) while effectively preventing GLM leakage and ensuring electrical insulation. Tan et al. [32] infiltrated an eutectic Ga-based alloy into a Ni-plated Cu mesh to produce a high-performance TIM, which led to a significant temperature reduction in chip cooling tests. Kim et al. [33] enhanced the thermal conductivity of GLMs by adding Cu flakes, finding that 5 wt% Cu offered the optimal balance between high thermal conductivity and low thermal contact resistance, thus improving its effectiveness as a TIM. Kuang et al. [34] fabricated a high-performance TIM by infiltrating GLMs into Cu nanowire arrays, achieving an extremely low total thermal contact resistance of 3.20 mm^2^∙K/W, which is 86.3% lower than that of commercial thermal grease.

As listed in the typical studies of GLMs applied as TIM shown in Table 1, the primary challenges center on achieving and maintaining low interfacial thermal resistance [36]. A fundamental barrier is the native Ga_2_O_3_ oxide layer, which causes poor wettability (~145° contact angle) and creates a phonon-conducting interface mismatched with the electron-dominated heat transfer in metals, resulting in weak van der Waals bonding and high thermal resistance [30]. Interfacial engineering can dramatically improve wetting, bonding strength and heat transport efficiency. One key method is the in situ formation of a CuGa_2_ IMC layer, achievable through processes like HCl treatment [30]. The overall role of CuGa_2_ remains complex because studies indicate that its spontaneous formation can increase electrical resistivity and may contribute to long-term corrosion [37]. Beyond these issues, the practical implementation of GLM–TIMs faces the ongoing challenge of ensuring long-term reliability against leakage and substrate corrosion, as well as balancing the surface modifications for electrical insulation [27].

#### 2.1.2. GLMs Applied as Heat Transfer Coolant

**Figure 3:**
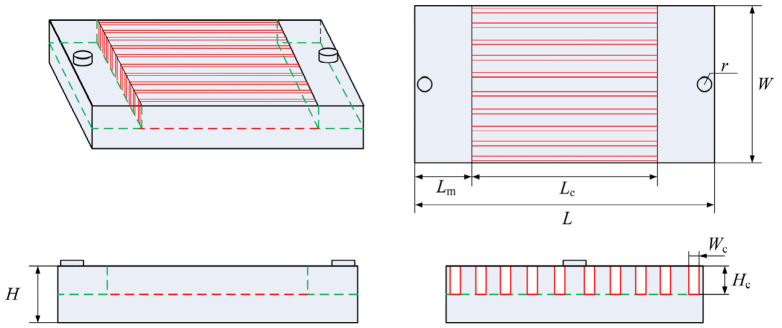
The structure of the liquid metal-based microchannel heat sink [52].

Microchannel cooling has emerged as an effective strategy to address the thermal management challenges of high heat-flux electronics, owing to its large surface-area-to-volume ratio and reduction in thermal boundary layers [44]. As the power density of electronic devices continues to rise, conventional liquid coolants such as deionized water increasingly struggle to meet the thermal requirements at heat-flux levels exceeding 500 W·cm^−2^ [45]. Their inherently limited thermal conductivity and boiling point often lead to flow instabilities, local boiling and even thermal failure, thereby constraining the advancement of microchannel cooling technologies. In this context, GLMs can offer significant promise as the next-generation coolants since they combine the advantages of metal and fluid. Liu et al. [46] proposed the use of GLMs as a coolant for effective chip cooling, which exhibited thermal conductivities of approximately 30 W·m^−1^·K^−1^, which is several tens of times greater than water. In addition, the boiling points of GLMs are generally above 2000 °C, which enable stable single-phase operation across a remarkably wide temperature range [47]. Their chemical stability, non-flammability and compatibility with common microfabrication materials further facilitate intimate interfacial contact and reduce thermal contact resistance. These unique attributes have driven growing interest in GLM-based microchannel cooling, positioning GLMs as a transformative solution to overcome long-standing performance bottlenecks in microchannel cooling, as shown in Figure 3.

Previous studies have demonstrated the potential of GLMs as a heat transfer coolant. Zhang et al. [48] proposed an optimized GLM-based microchannel heat sink and conducted a comparative study against a water-cooled configuration. The results showed that the EGaInSn (eutectic Ga-In-Sn) coolant required a larger channel width than the water-based system and sustained a maximum heat flux of 1504 W·cm^−2^. It significantly exceeded the 1003 W·cm^−2^ achieved by the water-cooled counterpart, thereby demonstrating the performance advantages of GLM-based coolants in high heat-flux applications. In addition, Tawk et al. [49] compared the thermal resistance of Cu–water and Cu–GaIn microchannels. Their study demonstrated that the thermal resistance of GLMs-based microchannels was reduced by 48% compared with water at identical pumping power, highlighting the superior energy efficiency of GLM coolants. 

Reducing hydraulic resistance, increasing flow velocity and minimizing thermal interfacial resistance are central to enhancing the thermal performance of GLM-based microchannel systems. Building on these principles, a series of subsequent studies has proposed and experimentally verified various design strategies, further underscoring the potential of GLMs in high heat-flux thermal management. Deng et al. [50] developed a two-stage multichannel GLM-based cooling system tailored for chip arrays, which investigated the heat sink and heat source temperatures of the GLM-based and pure water-cooling system with a heat-flux densities of 100 W/cm^2^. Using a GLM-based coolant in mini-channels with a width of 9.5 mm, the system achieved a convective heat transfer coefficient of approximately 20,000 W·m^−2^·K^−1^ at 50 W heating power and a flow rate of 3 mL·s^−1^. As it is three times that of comparable water-based systems, the superior cooling capability of GLMs under high heat-flux conditions was highlighted. Zhang et al. [51] proposed an innovative manifold-microchannel heat sink architecture employing GLMs as the working fluid. Experimental characterization revealed that the optimized flow distribution significantly enhanced the convective heat transfer coefficient to the order of 3.5 × 10^5^ W·m^−2^·K^−1^. This structure enabled stable dissipation of heat loads approaching 1000 W·cm^−2^, which showed much better performance than conventional water-cooled manifold systems. Collectively, these advances demonstrate the exceptional heat dissipation capability of GLMs and illustrate how tailored channel designs can further elevate their performance, which established a solid foundation for future power electronics thermal management.

Typical works of GLMs applied as microchannel coolant are summarized in Table 2, which exhibit that there are still challenges related to their unfavorable fluidic properties and interfacial behavior [53]. High viscosity and density lead to significant flow resistance and pumping power requirements, making larger channel geometries (>1.5 mm) more practical [54]. Furthermore, poor wetting on common substrate (e.g., Ni) can cause high interfacial thermal resistance, while the need for compatible coatings and complex system optimization to manage extreme pressure drops (>3 MPa) presents substantial technical difficulties [44,49].

### 2.2. GLMs Applied in Electrical Interfaces

#### 2.2.1. Solidified GLMs for Electrical Interconnection

Low-temperature interconnection is desirable in power electronics packaging to mitigate CTE mismatch when integrating dissimilar materials [55]. As room temperature liquids, GLMs can be solidified via the TLPB process at significantly lower temperatures by serving as the reactive metallic solvent [56]. For instance, Liu et al. [57] demonstrated that eutectic Ga-Sn (EGaSn) alloy can form solid joints with Au-coated Cu substrates at room temperature through the formation of CuGa_2_. Synchrotron X-ray fluorescence microscopy revealed that trace amounts of Au from the coating migrate to the top of the interfacial CuGa_2_ layer. 

**Figure 4:**
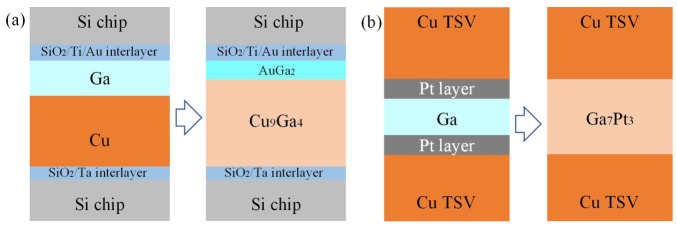
(**a**) Schematic of the interface prepared for Cu/Ga TLPB process; (**b**) schematic cross-sections of the Cu/Pt/Ga/Pt/Cu sandwich couples.

As shown in Figure 4a, Joerg et al. [58] performed the Cu/Ga TLPB process at room temperature followed by annealing, which triggered a phase transition from CuGa_2_ to Cu_9_Ga_4_ at the interface. This transformation significantly enhanced the mechanical strength of the bond, achieving shear strengths up to 90 MPa after treatment at 90 °C for 200 h. Lin et al. [59] developed a Ga-based soldering method using Pt under-bump metallurgy (UBM) to create ductile solid-solution joints for Cu-Cu interconnection over a period up to 24 h (Figure 4b). By adjusting the relative amounts of Ga and Pt, the process can produce either a desirable ductile face-cantered cubic (FCC) solid-solution joint with isolated precipitates when Ga significantly exceeds Pt, or a continuous brittle intermetallic layer when Ga is insufficient relative to Pt. These results demonstrate that GLMs can form TLPB joints at relatively low temperature with good mechanical properties. However, this process is time-consuming which usually needs dozens or even hundreds of hours. Therefore, it would be beneficial to develop interfacial reaction to accelerate this GLM-based TLPB process.

#### 2.2.2. GLMs for Fluidic Electrical Interconnection

**Figure 5:**
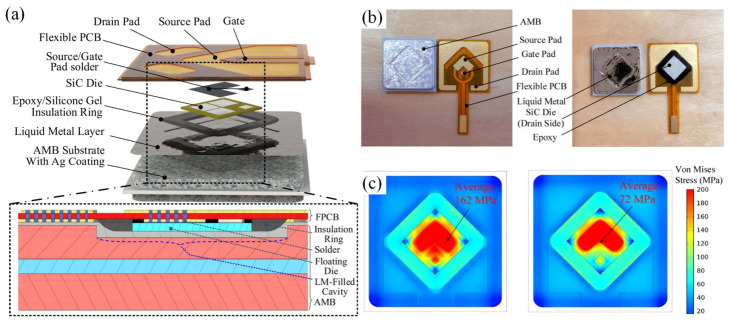
(**a**) Structure of the GLM-based SiC module; (**b**) prototype of GLM-based packaging and experimental setup; (**c**) thermomechanical stress comparison of the die and the substrate with solder-based packaging and GLM-based packaging [61].

Fluid-state electrical interconnection based on GLMs has shown potential in power electronics packaging due to its unique combination of metallic thermal conductivity and fluidic deformability. Fluidic interconnection can effectively alleviate thermal–mechanical stress between the die and substrate, thereby improving heat transfer and enhancing long-term reliability in high-power modules [60]. As exhibited in Figure 5, Liu et al. [61] demonstrated a GaInAg-based fluidic interconnection and floating die structure that minimizes thermomechanical stress by allowing self-adaptive movement of the die, achieving a stress by over 60% compared to conventional solder joints. Zhang et al. [24] reported a SiC half-bridge power module utilizing GLM interconnection, achieving low thermal resistance (0.08 K·W^−1^) and maintaining structural integrity under high-temperature cycling. Li et al. [62] further confirmed that GLM packaging for press-pack IGBT modules enhances both electrical interface uniformity and heat dissipation performance compared to the conventional Sn-based solders.

Table 3 presents representative research on GLMs applied as fluidic electrical interconnects. These applications face several key challenges, primarily concerning interfacial instability and material compatibility [63]. The spontaneous formation of resistive IMCs (e.g., CuGa_2_) at interfaces, especially with common conductors like Cu, degrades electrical conductivity and long-term reliability [64]. In humid environments, controlling wetting behavior and preventing corrosion are critical since poor adhesion or oxidation can increase contact resistance and cause failure [65]. Furthermore, while barrier coatings can mitigate reactions, they may also reduce conductivity or increase electrical resistance [64]. The inherent high surface tension and potential for component separation in pastes also pose challenges for achieving uniform and durable electrical interconnections [60].

Overall, GLM-based fluidic interconnections represent a transformative strategy for next-generation power electronics. They bridge mechanical compliance with superior heat conduction, supporting reduced interfacial stress while enabling ultrahigh heat-flux dissipation (>1000 W·cm^−2^). The electrical advantages of GLMs in power modules arise from their high electrical conductivity, which meets interconnection requirements. In addition, their unique fluidic nature enables adaptive deformation to form conformal contact with device surfaces, thereby reducing interfacial contact resistance effectively.

## 3. Properties of Regular GLMs and Interfacial Reaction with Cu

### 3.1. General Properties of Regular GLMs

Ga is a post-transition metal with a melting point of 29.76 °C and a viscosity of 1.99 cps, which is similar to water [71]. Its unusual behavior can be explained by its unique electronic structure, which features both covalent and metallic bonding, as well as the formation of Ga dimers that influence its phase changes. [72]. The CTE of Ga is approximately 18.3 × 10^−6^ K^−1^, which is close to Cu (16.9 × 10^−6^ K^−1^) [73]. GLMs have garnered attention in electronic packaging area due to their excellent thermal and electrical properties, liquid-state characteristics and low viscosity [74]. The development of low-temperature GLM-based TLPB processes effectively reduced energy consumption and minimized the risk of damage to heat-sensitive electronic components [75].

In addition to pure Ga, certain Ga-based alloys exhibit lower melting points, broadening their applications range [76]. Alloying Ga with metals such as In, Sn, Zn, Cd or Pb enables the formation of eutectic alloys with lower melting points [77]. For instance, the eutectic Ga-In alloy (EGaIn), composed of 75.5 at% Ga and 24.5 at% In, has a melting point of 15.4 °C [71]. The melting point of EGaIn can be tuned by varying the addition ratio of Ga and In. Another example is EGaInSn (eutectic Ga-In-Sn), which has a melting point of 13.2 °C [71]. The physical properties of Ga, EGaIn and EGaInSn are listed in Table 4.

As electronic devices advance toward higher power density, frequency and temperature conditions, the Joule heating and switching losses generated during operation have increased significantly, leading to a sharp rise in die junction temperatures [78]. In this context, the thermal management performance of interconnect materials has become a critical bottleneck that limits the reliability and lifetime of electronic devices. GLMs, including Ga, EGaIn and EgaInSn, exhibit excellent thermal conductivity for efficient heat transfer [79]. Furthermore, their liquid-state properties help mitigate thermal–mechanical stresses induced by non-uniform thermal fields. By suppressing failure modes such as thermal fatigue and electromigration, GLMs ensure the long-term operation of electronic devices in harsh environments.

GLMs are viscous fluids that retain metallic properties, with positive ions and free electrons that remain mobile, resulting in high electrical conductivity. Seungho et al. [80] employed first-principle molecular dynamics simulations along with the Kubo–Greenwood (K-G) and Ziman–Faber (Z-F) theoretical approaches to calculate the electrical conductivity of liquid Ga, EGaIn and EGaInSn. The results demonstrated that the incorporation of In and Sn into Ga to form eutectic alloys increases atomic structural disorder, leading to enhanced electron scattering, shortening of the electron mean free path, and a consequent reduction in the electrical conductivity of GLMs.

### 3.2. Interfacial Reaction Between GLMs and Cu-Based Substrates

In a standard module, the substrate is typically structured in a sandwich-like configuration. Its core consists of a thick and electrically insulating ceramic sheet, which is clad on both sides with conductive layers to form the circuit [81]. Cu is the most common upper conductor metal in a substrate due to its excellent electrical and thermal conductivity [82]. Therefore, it is the most possible that GLMs would form the thermal or electrical interfaces with Cu. However, Cu is easily oxidized when served in a humid or oxygen-containing environment, making a protective layer necessary [83]. For instance, Cu can dissolve quickly in Pb-free molten solders, resulting in an unstable microstructure that poses a potential reliability risk to bonded structures [84].

**Figure 6:**
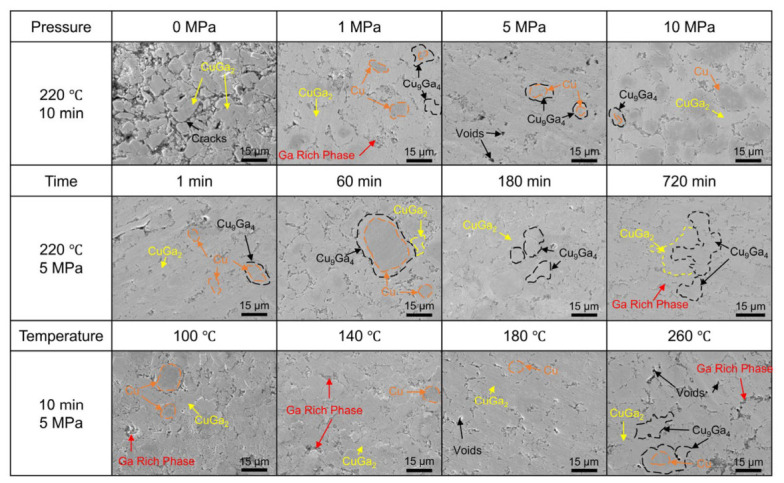
Micrographs of GLM-Cu TLPB joints under different bonding parameters [89].

Ga can dissolve in the FCC Cu-rich phase with a solubility of up to 20 at.%, leading to the formation of several IMCs over a wide temperature range. Consequently, GLMs have emerged as promising candidates for enabling TLPB at low processing temperatures [85]. This approach facilitates the formation of full-IMC structures that exhibit excellent mechanical and thermal stability under high-temperature service conditions [86]. Within the temperature range of room temperature to 300 °C, the interfacial reaction between Ga and the Cu substrate primarily involves the formation of two IMC phases including *θ*-CuGa_2_ and γ_3_-Cu_9_Ga_4_. At temperatures between 150 and 240 °C, the reacting interface exhibits a layered structure of Cu/γ_3_-Cu_9_Ga_4_/*θ*-CuGa_2_/liquid Ga, where γ_3_-Cu_9_Ga_4_ forms a thin planar layer and *θ*-CuGa_2_ develops a thick scallop-like morphology [87]. The *θ*- CuGa_2_ phase decomposes at approximately 254–264 °C with the reaction 9*θ*-CuGa_2_→γ_3_-Cu_9_Ga_4_+14Ga. With increasing reaction time or temperature, the formation of γ_1_-Cu_9_Ga_4_ and γ_2_-Cu_9_Ga_4_ phases may also occur [88]. When the temperature increases to 280–300 °C, *θ*-CuGa_2_ becomes thermodynamically unstable and decomposes, leaving only the scallop-shaped γ_3_-Cu_9_Ga_4_ phase at the interface [86]. Notably, Cu dissolves preferentially at defect-rich sites, forming basin-type morphologies where *θ*-CuGa_2_ nucleates. Thereby, IMC detachment and subsequent diffusion-limited growth then lead to a continuous *θ*-CuGa_2_ layer and γ_3_-Cu_9_Ga_4_ formation [79]. The typical microstructures of GLM-Cu TLPB joints composed by CuGa_2_ and Cu_9_Ga_4_ IMCs are shown in Figure 6 [89].

**Figure 7:**
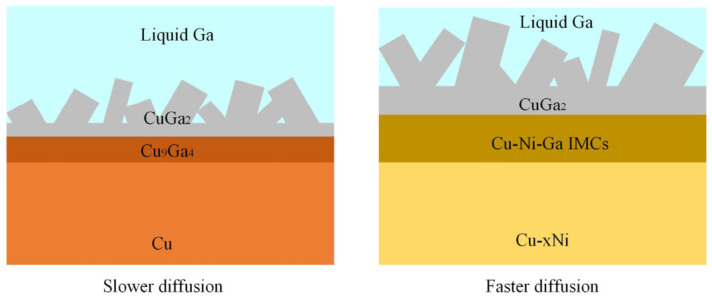
Interfacial reactions between Ga and Cu or Cu-xNi substrate.

Compared with traditional Sn-based IMCs, CuGa_2_ exhibits lower thermal expansion anisotropy, hardness and elastic modulus, making it is more favorable for micro-solder joints due to improved interconnection reliability [86]. However, the slow reaction kinetics between pure Cu substrates and Ga significantly constrain their practical applicability. Liu et al. [90] demonstrated that Ni alloying alters the interfacial reaction pathway in Cu. During the reaction between Ga and a Cu-10Ni substrate, not only CuGa_2_ is formed; a nanocrystalline Ga-5Ni layer and a Ni-substituted phase are formed. The presence of Ni markedly accelerates IMC growth, with the rate-controlling mechanism transitioning from volume-diffusion-dominated growth to a hybrid mechanism governed by both interfacial reaction and lattice diffusion [90], as illustrated in Figure 7. Systematic investigations of Cu-xNi substrates containing 2–14 wt% Ni revealed that the Cu-6Ni composition yields the highest IMC growth rate in Cu-xNi/Ga/Cu-xNi joints. In contrast, excessive Ni content (e.g., 14 wt%) promotes the formation of additional phases such as Ga_7_Ni_3_ [91].

**Figure 8:**
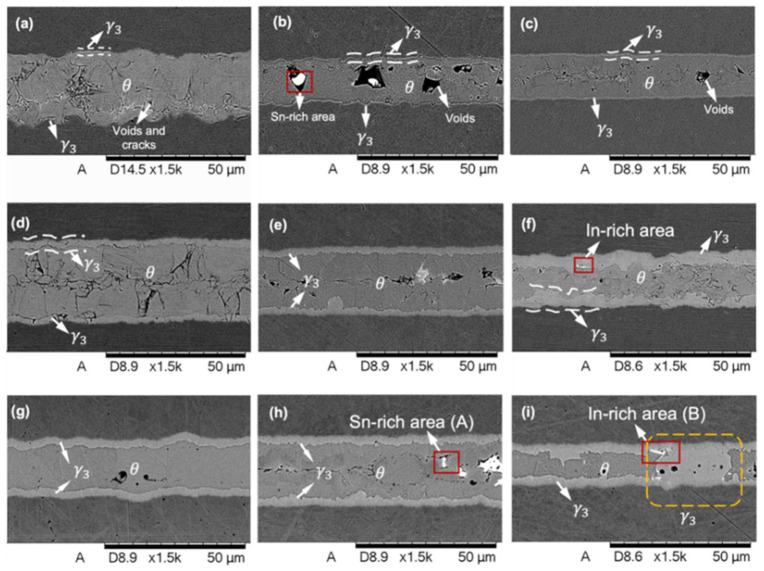
The cross-section SEM images of Cu-Cu joints after 90 min reaction: (**a**) Cu/Ga/Cu, (**b**) Cu/GaSn/Cu and (**c**) Cu/EGaIn/Cu at 150 °C, (**d**) Cu/Ga/Cu, (**e**) Cu/GaSn/Cu and (**f**) Cu/EGaIn/Cu at 220 °C and (**g**) Cu/Ga/Cu, (**h**) Cu/GaSn/Cu and (**i**) Cu/EGaIn/Cu at 220 °C, 1 MPa [85].

EGaIn (Ga-24.5% In, eutectic point 15.5 °C) and EGaInSn alloy (Ga-21.5% In-10% Sn, eutectic point 13.2 °C) [60] are the representative Ga-based alloys with low melting point and high fluidity. Chen et al. [85] investigated the application of TLPB using pure Ga, eutectic GaIn and GaSn with Cu. The study found that the reaction between EGaIn and Cu primarily forms the interfacial phases θ-CuGa_2_ and γ_3_-Cu_9_Ga_4_. Atoms did not incorporate into these IMCs but segregated between their grains and formed a thin liquid layer. The presence of the In element accelerated Cu atom diffusion and promoted the formation of the γ_3_-Cu_9_Ga_4_ phase. The porosity in the joints was reduced and the joint reliability was thereby enhanced. The final Ga-based IMC joints comprise γ_3_ and θ layers, with residual Sn- and In-rich regions segregated at the IMC interfaces. Figure 8 presents cross-sectional SEM images of Cu/Ga/Cu, Cu/GaSn/Cu, and Cu/EGaIn/Cu joints following the TLPB process [85]. Although the mixing enthalpy of Sn-Ga is comparable to that of Cu-Ga, the diffusion barrier effect of the θ-CuGa_2_ layer suppresses Cu-Sn reactions. Consequently, θ-CuGa_2_ remains the dominant interfacial product, which is accompanied by limited Sn segregation between IMC grains [85]. Gao et al. [92] examined liquid–solid interfacial reactions in EGaInSn/Cu couples under isothermal and non-isothermal conditions (140–310 °C), emphasizing the influence of temperature and thermal gradients on CuGa_2_ formation. Their results indicated that tetragonal CuGa_2_ is the predominant product below 310 °C. Its growth mechanism and preferred orientation change markedly with temperature. At a fixed hot-side temperature, thermal gradients have little effect on CuGa_2_ morphology but can enhance directional Cu diffusion, thereby accelerating IMC growth.

## 4. Regulation Strategies of Packaging Interfaces

### 4.1. Acceleration Methods for GLM-Based TLPB Process

**Figure 9:**
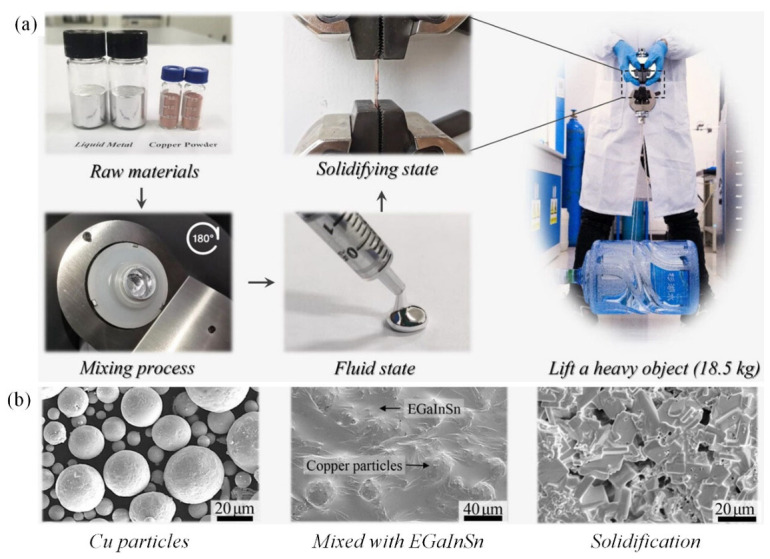
(**a**) EGaInSn/Cu composite preparation and transition phenomenon; (**b**) SEM images of EGaInSn/Cu composites [9].

TLPB is a promising interconnect method for WBG semiconductor power devices. However, the wide application is limited by the long processing time required to form the full-IMC joints [93]. Increasing the contact area is a potential strategy to accelerate TLPB. By introducing materials with high specific surface area, atomic diffusion and IMC formation are promoted. In particular, open-cell metal foams are used as reinforcing phases to enhance interfacial reaction due to their three-dimensional continuous network structure. This structure shortens the diffusion distance and increases the grain boundary diffusion pathways, thereby reducing the time needed to form the full-IMC joint. For instance, for a regular Cu/Sn (6 μm)/Cu structure to form a full Cu-Sn IMC at 260 °C, it requires 120 min [94]. In comparison, the TLPB processing time for a Sn/Cu foam composite solder is shortened by 96%. As shown in Figure 9, Zheng et al. [9] achieved rapid TLPB by mixing EGaInSn with micro Cu particles. After solidification at room or moderately elevated temperatures (20−60 °C), the composite formed a robust bond with Cu substrates [9].

In addition, the use of metal form or mesh as an enhancement phase refines IMC grains, thereby promoting their mechanical properties [95]. The large Cu_6_Sn_5_ grains in the middle area underwent typical brittle fracture. By applying Cu foam/SAC305 composite solder, the shear strength of the joints was significantly increased by 27% in a shorter time [96]. This improvement in strength and ductility of the TLPB joints is attributed to the combined effect of the ductile three-dimensional continuous Cu-foam framework and fine-grain strengthening from Cu–Sn IMCs [95]. Compared with the pure Sn solder joints, the foam Ni/Ag composite solder with 7075 Al [97] and 2024 Al alloy [98] has significantly increased the shear strength by 37% and 344.8%, respectively. Ni foam improves the tensile strength of SAC105 and SAC105-0.3Ti solder joints, which are, respectively, increased by 65.5% and 62.5% compared with the base solder joints [99]. At relatively low temperatures, the interfacial reaction between GLMs and Cu is relatively slow. To achieve the desired bonding strength, the reaction time at low temperatures may need to be prolonged, potentially lasting tens of hours [9]. In particular, dense oxide films can hinder the wetting of GLMs and reduce the efficiency of interfacial reactions in certain scenarios, which are the potential challenge for low-temperature TLPB [100]. 

**Figure 10:**
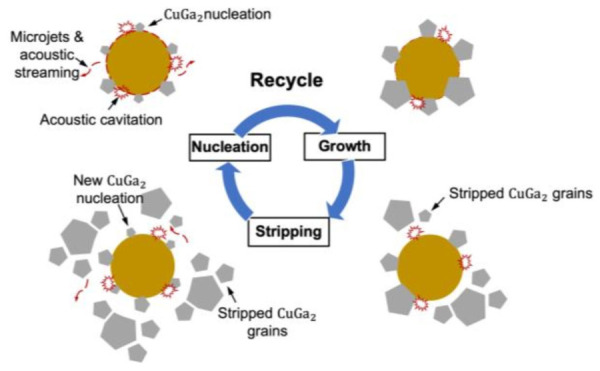
Schematic diagram of Ga-Cu IMC growth and strip on Cu particle surface during the ultrasonic-assisted TLPB process [15].

Ultrasonic technology has long been used for rapid wire bonding, as ultrasonic waves can reduce the temperature required for plastic deformation [101]. Ultrasonic-assisted soldering has also been developed, relying on the ultrasonic acoustic cavitation effect that appears when ultrasonic signal is propagated in the liquid medium [102]. This phenomenon can generate numerous cavitation bubbles within the molten solder. The subsequent collapse of these bubbles creates high-speed liquid jets that continuously impinge on the solid surface, effectively removing surface oxide layers and improving solder wettability [103]. Furthermore, cavitation bubbles collapsed near the reaction interface, producing microjets and acoustic streaming, and releasing localized heat and enhancing mass transfer. These effects collectively accelerate interfacial reactions. With continuous ultrasonic application, the cyclic formation and collapse of cavitation bubbles disrupt and detach the IMC grains that have formed on the reaction surface. This allows the liquid solder to contact the freshly exposed substrate through the fractured IMC layer, thereby shortening the diffusion path across the reaction interface. Throughout this process, the nucleation, growth, and detachment of IMCs occur cyclically, resulting in finer-grained IMCs and consequently superior mechanical properties, as shown in Figure 10 [15].

Ultrasonic vibration represents a promising approach for shortening the GLM-based TLPB process. In related studies, Zhao et al. [104] reported that the efficiency of the solid Al dissolution in liquid Sn with ultrasound was improved remarkably, and the activation energy of dissolution was reduced markedly. Li et al. [100] found that the bonding speed of 7075-Al alloy joints using Ni-mesh-reinforced SAC305 composite solder could be accelerated with appropriate ultrasonic treatment, resulting in improved shear strength. Liu et al. [2] found that the TLPB assisted by ultrasonic vibration can accelerate the interfacial reactions between Zn-5Al and Cu or Ni substrate under ambient conditions. The uniform full-IMC joints can be formed within 1 min and 3 min on Cu and Ni substrate, respectively, without flux applied. Chen et al. [15] demonstrated that the ultrasonic vibration considerably accelerates the bonding process with Cu-Ga paste, enabling the formation of full-IMC joints with a uniform microstructure within 5 min under low-temperature conditions.

### 4.2. Decelerating Methods for GLM Interfacial Reaction

Metallization is one of the most popular methods to decelerate the interfacial reaction, offering a wide choice of finish materials and manufacturing routes [105]. It is commonly applied for enhancing solderability and preventing substrate dissolution and oxidation [106]. Ni coatings with thicknesses of several micrometers are often employed as a diffusion barrier to protect the underlying Cu substrate, as Ni reacts significantly slower than Cu during soldering [107]. Ag and Au finishes are used to prevent surface oxidation, which also provide excellent chemical stability for long-term storage [108]. The most common coating structures include immersion Ag (ImAg) [109], electroless Ni/immersion Au (ENIG) [110] and electroless Ni/immersion Ag (ENImAg) [111]. 

**Figure 11:**
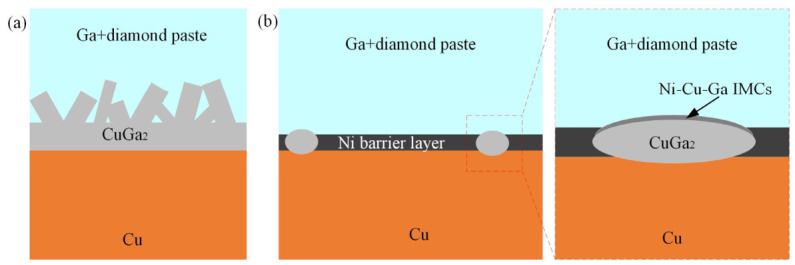
(**a**) Diffusion between Ga + diamond paste and Cu substrates; (**b**) effect of Ni-based barrier layer.

Metallization with Ni is widely employed as the diffusion barrier layer for GLM-based fluidic interconnections. In ENIG structures, the electroless Ni layer suppresses Ga infiltration and delays IMC growth at the interface. When liquid Ga alloys contact Ni, the reaction typically forms Ga-Ni IMCs such as Ga_7_Ni_3_, which grow with relatively slow kinetics and exhibit a self-limiting characteristic that restricts further diffusion [112,113]. These IMCs evolve through reaction-controlled or mixed growth mechanisms at moderate temperatures, resulting in significantly lower dissolution rates than Cu-Ga systems. However, Ni metallization can be gradually consumed by GLMs at 160 °C [114]. Electrodeposited Ni-W coatings exhibit negligible degradation when exposed to Ga-Sn-Zn alloys below 250 °C, maintaining a dense and continuous microstructure even after extended aging [115]. Such behavior indicates that alloying elements like W effectively reduce Ga diffusivity and enhance the thermal robustness of the barrier layer. Compared with the Ni layer, the Au cap in ENIG does not provide substantial reaction resistance. Liquid Ga reacts rapidly with Au to produce a single AuGa_2_ layer with fast growth kinetics, making Au prone to spalling and local delamination during prolonged contact [116]. As shown in Figure 11, electroless Ni or Ni-P coatings effectively block Ga wetting and suppress interfacial dissolution, ensuring stable thermal resistance during repeated thermal cycling [43]. Overall, pure Ni metallization could effectively decelerate GLM-induced interfacial reactions at relatively low-temperature, novel metallization; protection methods are still demanding at elevated thermal conditions.

**Figure 12:**
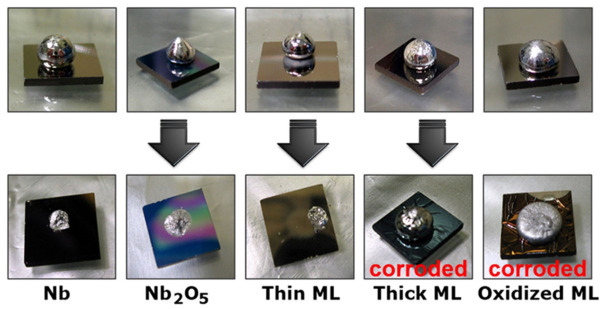
The liquid Ga-resistant test with Nb-based coatings [118].

Refractory metals, such as Nb and Ta, have been regarded as potential diffusion barrier layers for GLM interconnects due to their high melting points and chemical stability [117]. A 70 nm Nb layer can withstand Ga for 30 h at 200 ℃, showing better performance than regular Ni barrier layers in high-temperature conditions [118]. In multilayer Nb/NbO_x_ coatings, Ga diffusion was still observed at elevated temperatures, indicating that although the oxide layer provides a better barrier effect than pure Nb coatings, it still cannot completely prevent interfacial diffusion. This behavior is illustrated in Figure 12, which shows the interactions between Ga and different Nb/NbO_x_ coatings [118]. The Ga-Nb phase diagram shows that Ga exhibits finite solubility in Nb at high temperatures, which can lead to interfacial degradation when the barrier layer is thin. Ga-Nb intermediate phases are thermodynamically possible, and although their formation requires elevated temperatures, the presence of Ga-rich liquid accelerates the local dissolution of Nb and limits its long-term reliability as a barrier material [119].

Ta exhibits superior resistance to Ga due to its very low Ga solubility and the sluggish formation of IMCs. Under high-pressure and high-temperature conditions, Ta remains essentially inert to liquid Ga up to ~500 °C, showing negligible lattice expansion and no detectable dissolution [120]. Only at temperatures above 600−700 °C does Ta begin to form Ga_3_Ta, and the reaction rate remains very slow compared to other metal–Ga interfaces. This stability originates from Ta’s strong bonding and limited mutual solubility, making it one of the most Ga-resistant refractory metals. However, thin Ta coatings may not replicate the same level of protection as bulk Ta. Studies on nanoscale barrier reveal that 100 nm Ta layers do not effectively block Ga migration under thermal cycling, as Ga can penetrate through grain boundaries or microdefects [121]. Therefore, although Ta is intrinsically more chemically inert compared to Ga and Nb, structural optimization remains necessary before it can serve as a reliable protection layer in fluidic interconnections.

### 4.3. Regulations of Thermal and Electrical Properties at Interfaces

**Figure 13:**
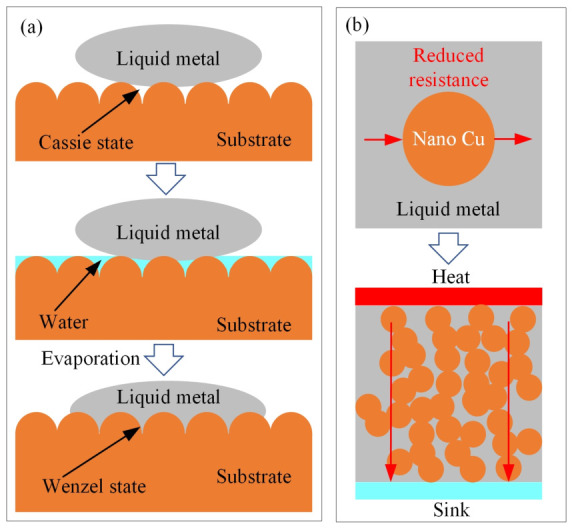
(**a**) GLM wetting transition from the Cassie to Wenzel state; (**b**) GLM thermal properties improved by high thermal conductivity fillers.

To regulate the interfacial thermal properties of GLMs, several effective strategies have been promoted by enhancing wetting, forming conductive intermetallic layers and constructing composite thermal pathways. For instance, as shown in Figure 13a, infiltration driven by water evaporation can transition GLM wetting from the Cassie to Wenzel state on microstructure Cu, substantially increasing the contact area and reducing interfacial thermal resistance [122]. Chemically induced in situ formation of a CuGa_2_ intermetallic layer through HCl treatment will replace the insulating Ga_2_O_3_ oxide, switching heat transfer from phonon-dominated to more efficient electron-dominated transport and greatly improving interfacial thermal conductivity [30]. Additionally, as shown in Figure 13b, compositing GLMs with high thermal conductivity fillers, such as adding 4 vol% Cu nanoparticles, can raise the thermal conductivity of the TIM by ~180% without undermining fluidity [38]. Furthermore, employing pre-structured materials like vertically aligned graphene films or Cr-coated diamond particles helps to establish continuous three-dimensional heat conduction networks, further lowering interfacial thermal resistance and improving stability under thermal cycling [36,39]. In summary, these approaches demonstrate that proper interfacial engineering and material design are promising to optimize the thermal performance of GLMs.

To regulate the interfacial electrical properties of GLM interconnects, key strategies focus on suppressing undesirable interfacial reactions and establishing stable, low-resistance contacts. Effective diffusion barrier layers like Ni-Co or electrodeposited Ni-W coatings can successfully block the formation of high-resistivity intermetallic compounds (e.g., CuGa_2_) and maintain stable interfaces at elevated temperatures [123]. Alternatively, promoting the controlled formation of a thin, continuous IMC layer like CuGa_2_/Cu_9_Ga_4_ at the interface can itself act as a protective barrier, resulting in a low and stable contact resistance (~2.5–10 mΩ) during long-term service conditions [63]. Furthermore, surface treatments such as HCl vapor can significantly improve initial wettability and interfacial bonding, enabling reliable electrical performance comparable to traditional solders [69]. The selection of substrate crystallography is critical because interfaces with higher adhesion energy, such as Cu(111)/CuGa_2_, provide greater mechanical and electrical stability [70]. Therefore, the choice of regulation methods involves a balance among long-term resistance stability, corrosion inhibition and compatibility with the operating environment.

## 5. Summary

This study provides a comprehensive review on the properties, applications and interfacial regulation strategies with GLMs for power electronics integration. Certain applications and advantages of GLMs in terms of serving as thermal and electrical interfaces are elaborated based on their unique properties, where potential technical challenges are yet to be addressed. Given the actual needs in the applications, potential interfacial regulation strategies can be proposed to fulfill the future reliability requirement due to increasing power density. The key conclusions can be summarized as follows.

(1) Owing to their high thermal and electrical conductivity and low melting point, GLMs offer various advantages for thermal management and low-temperature bonding, which are potentially viable for the integration of high-power modules. However, purposely altering or regulating the interfacial interactions for a specific application can ultimately govern long-term reliability in service and operation, which is primarily determined by the interactive interfacial reactions between GLMs and metallic substrates, where a surface coating may be necessary to achieve the goals.

(2) For thermal management, the primary cause of failure is attributed to the interfacial reactions in the applications of GLMs as TIMs or heat transfer. The Cu substrate can react with Ga and Ga-based alloys at room temperature, resulting in the formation of IMCs, which can lead to interfacial failure. Protective Ni-based metallizations such as ENIG or Ni-W layers are proven to be effective in retarding the interfacial reactions to certain extents, which, hence, improves reliability. However, the refinery metals such as Nb and Ta coatings are the most desirable in enabling the sufficient protection of the underlying Cu substrate at elevated temperatures (>200 °C), which is likely to occur under high-power density conditions.

(3) The main challenges of GLMs applied for thermal management centered on balancing interfacial performance with fluidic properties. As a coolant, the high viscosity and density of GLMs lead to significant flow resistance and pumping power consumption, limiting their application in smaller channels, while their poor wettability on common substrate materials introduces high thermal resistance, and system optimization is often limited by high pressure drops. As a TIM, their high interfacial thermal resistance mainly stems from poor wettability caused by the Ga_2_O_3_ surface oxide layer and the phonon–electron heat carrier mismatch. Although interface modification (e.g., forming a CuGa_2_ IMC layer) can significantly improve wettability and heat transfer efficiency, the effect of CuGa_2_ formation on thermal conductivity remains debated. Some studies suggest it increases electrical resistance and may promote corrosion, while others demonstrate that it enables efficient electron-dominated heat transfer. Furthermore, long-term reliability issues like substrate corrosion and leakage, as well as the complexity and compatibility demand of interfacial modifications, pose key challenges for both applications.

(4) For electrical connections using GLMs, both liquid-state and solid-state interconnection can be applied, subject to the specific applications. Interconnects via liquid GLMs can effectively decouple the interfacial thermal stress induced by CTE mismatch during the cycles of operation, but preventing the leakage of confined GLMs at the interfaces is critical to ensuring enhanced electrical conductivity. The TLPB process can be a tangible route of forming the interconnects, with GLMs reacting with metal substrate at a low temperature, thereby creating a full-IMC joint which is able to serve under a high-temperature regime. However, TLPB at low temperatures can be extremely time-consuming. To reduce the interfacial reaction time, it is practical to accelerate the inter-diffusion in TLPB processes by increasing the reactive contact area with GLMs by incorporating a metal (e.g., Cu) foam or mixing GLMs with micro/nano-metal particles. Ultrasonic vibration can also be applied to assist the inter-diffusion, thus speeding up the TLPB interfacial reactions, as the result of enhanced wettability and atomic inter-diffusion due to the breakdown of surface oxides resulted from effective acoustic interruptions. 

(5) The challenges of GLMs were applied as fluidic conductive interconnects, primarily due to interfacial instability and material compatibility issues. Their inherent poor wettability caused by the native Ga_2_O_3_ layer leads to high and unstable contact resistance, which is further worsened in high-humidity environments. The spontaneous formation of IMCs like CuGa_2_ improves interfacial bonding but often degrades electrical conductivity due to their higher resistivity. Long-term reliability is threatened by the corrosion of substrate metals (especially Cu), potential component separation within the GLM paste, and failure mechanisms like GLM pump out. Furthermore, while barrier coatings such as Ni-W, diamond or noble metals can mitigate interfacial reactions, they also introduce limitations in electrical conductivity, thermal performance and cost. Consequently, designing a fluidic electrical interface that is stable, low-resistance and durable remains a persistent engineering challenge.

## Figures and Tables

**Table 1:** Typical studies of GLMs applied as TIMs.

GLMs Investigated	Interfacial Materials with GLMs	Main Conclusion	Ref.
Ga-based alloy	Cr-coated diamond particles	Cr-coated diamond particles form a 3D thermal network with LM; pressure-induced exudation reacts with Cu to form a low thermal resistance interface (0.206 K·mm^2^/W).	[36]
EGaIn	Cu	Heat treatment forms a stable CuGa_2_/In(Ga)/LM interfacial structure, improving bond and inhibiting leakage.	[27]
Ga	Cu	Cu-C alloys exhibit superior corrosion resistance to liquid Ga compared to pure Cu, especially at elevated temperatures.	[37]
EGaInSn	Cu nano particles	Adding 4 vol% Cu nanoparticles increases thermal conductivity by ~180% to 64.8 W·m^−1^·K^−1^ without compromising fluidity.	[38]
Carbon nanotube (CNT)-coated GLM particles	Vertically aligned graphene film (VAGF)	VAGF-based composite significantly lowers LED operating temperature (by 42 °C) and maintains stable performance over many thermal cycles.	[39]
EGaIn	Porous copper	Electrochemical wetting in NaOH enables rapid LM infusion into porous Cu, forming a super-wetting CuGa_2_ coating.	[40]
Ga	Cu	HCl treatment removes oxides, enabling in situ formation of a CuGa_2_ interlayer which dramatically improves wetting and electron-dominated heat transfer at the interface.	[30]
Ga	Cu	Surface-modifying copper particles with 3-chloropropyltriethoxysilane (CPTES) enhances interfacial thermal conductance and acts as a diffusion barrier, boosting composite thermal conductivity to 65.9 W·m^−1^·K^−1^.	[41]
Ga	CuGa_2_	Abnormal wetting of Ga on CuGa_2_ is driven by strong metallic bonding due to favorable electron exchange, not traditional wetting theory.	[42]
Ga	Cu and Ni	Charge redistribution simulations indicate the formation of polar chemical bonds at the Cu-Ga interface, explaining enhanced bonding.	[43]

**Table 2:** Typical studies of GLMs as microchannel coolants.

GLMs Investigated	Interfacial Materials with GLMs	Main Conclusion	Ref.
Ga-based alloy	Cr-coated diamond particles	Cr-coated diamond particles form a 3D thermal network with LM; pressure-induced exudation reacts with Cu to form a low thermal resistance interface (0.206 K·mm^2^/W).	[36]
EGaIn	Cu	Heat treatment forms a stable CuGa_2_/In(Ga)/LM interfacial structure, improving bond and inhibiting leakage.	[27]
Ga	Cu	Cu-C alloys exhibit superior corrosion resistance to liquid Ga compared to pure Cu, especially at elevated temperatures.	[37]
EGaInSn	Cu nano particles	Adding 4 vol% Cu nanoparticles increases thermal conductivity by ~180% to 64.8 W·m^−1^·K^−1^ without compromising fluidity.	[38]
Carbon nanotube (CNT)-coated LM particles	Vertically aligned graphene film (VAGF)	VAGF-based composite significantly lowers LED operating temperature (by 42 °C) and maintains stable performance over many thermal cycles.	[39]
EGaIn	Porous copper	Electrochemical wetting in NaOH enables rapid LM infusion into porous Cu, forming a super-wetting CuGa_2_ coating.	[40]
Ga	Cu	HCl treatment removes oxides, enabling in situ formation of a CuGa_2_ interlayer which dramatically improves wetting, bonding, and electron-dominated heat transfer at the Ga/Cu interface.	[30]
Ga	Cu	Surface-modifying copper particles with 3-chloropropyltriethoxysilane (CPTES) enhances interfacial thermal conductance and acts as a diffusion barrier, boosting composite thermal conductivity to 65.9 W·m^−1^·K^−1^.	[41]
Ga	CuGa_2_	Abnormal wetting of Ga on CuGa_2_ is driven by strong metallic bonding due to favorable electron exchange, not traditional wetting theory.	[42]
Ga	Cu and Ni	Charge redistribution simulations indicate the formation of polar chemical bonds at the Cu-Ga interface, explaining enhanced bonding.	[43]

**Table 3:** Typical studies of GLMs for fluidic electrical interconnects.

GLMs	Substrate/Contact Material	Main Conclusion	Ref.
EGaIn and EGaInSn	Au/Ni/Cu multilayer coating	EGaIn shows better long-term contact resistance stability than EGaInSn. Humidity accelerates interface degradation, requiring environmental sealing for reliability.	[65]
Ga mixed with Ga-oxide	Cu	LM’s liquid state provides immunity to thermomechanical stress, dramatically improving power cycling life. Corrosion remains a key challenge, and adding Ga-oxide increases viscosity for better shape control.	[60]
Ga mixed with Ga-oxide	Cu	GLM encapsulation improves thermal performance, tripling diode lifetime and reducing transient thermal impedance by 25% with low-temperature processing. However, reliability is limited by corrosion, pump out, intermetallic growth, and component separation.	[66]
EGaInSn	Ti substrate with B-doped diamond coating	The boron (B)-doped diamond coating exhibits superlyophobicity (contact angle >155°) and a low contact resistance of 0.84 Ω with Galinstan. Despite a resistivity three to four orders of magnitude higher than copper, it maintains stable electrical contact for 3000 min while serving as an anti-adhesion and diffusion barrier.	[67]
GaInAg	Ag coating	Replacing solder with a GLM die-attach layer decouples thermal strain, reducing chip stress by 56% while maintaining a comparable on-state resistance (15.98 mΩ vs. 15.53 mΩ for solder), offering a major mechanical benefit with minimal impact on electrical performance.	[61]
EGaIn	Cu, Sn, Ag electrodes	The stable IMC layer that forms at the EGaIn/Cu interface acts as an effective diffusion barrier while maintaining a low contact resistance of ~2.5–10 mΩ for 1000 hours at 100 °C. Cu is a reliable electrode for GLM interconnects, and its durability can be further enhanced through material and design improvements.	[63]
Ga	Pd (Palladium)	Pd serves as an effective wetting layer, with IMC growth kinetics shifting from interfacial reaction control to diffusion control as temperature increases.	[68]
EGaIn	Cr/Cu bilayer	HCl vapor treatment enables reliable electrical interfacing on Cr/Cu, with performance matching solder paste, as Ga diffusion is blocked by the Ni layer.	[69]
EGaInSn	Cu	The Cu(111)/CuGa_2_(001) interface exhibits the strongest adhesion and stability, but CuGa_2_ formation has minimal impact on overall interfacial electron transport.	[70]
EGaInSn	Electrodeposited Ni-W coating	The Ni-W coating effectively blocks the formation of high-resistivity IMC (e.g., CuGa_2_) at the GLM/Cu interface at temperatures up to 410 °C, with high electrical resistivity (~70–120 nΩ·m) and low thermal conductivity (~20–40 W/(m·K)).	[64]

**Table 4:** Physical properties of typical GLMs [28,49,73].

Material	Melting Point (°C)	Boiling Point (°C)	Viscosity (10^−7^·m^2^·s^−1^)	Specific Heat (J·kg^−1^·K^−1^)	Electrical Conductivity (10^6^·S·m^−1^)	Thermal Conductivity (W·m^−1^·K^−1^)
Ga	29.76	2403	3.24	397.6	3.7	29.4
EGaIn	15.5	2000	2.7	350	3.4	42.2
EGaInSn	13.2 ^α^	>1300	2.98	392	3.1	44.8

^α^ Note: The melting point of EGaInSn is often given as −19 °C which refers to the freezing temperature (which differs from the melting point due to supercooling).

## Data Availability

No new data were created or analyzed in this study. Data sharing is not applicable.

## Abbreviations

The following abbreviations are used in this manuscript:

GLMs	Ga-based liquid metals
TLPB	Transient liquid-phase bonding
TIMs	Thermal interface materials
WBG	Wide bandgap
IMC	Intermetallic compounds
CTE	Coefficient of thermal expansion
BGA	Ball grid array
UBM	Under-bump metallurgy
FCC	Face-cantered cubic
